# Claudin-1, -2 and -3 Are Selectively Expressed in the Epithelia of the Choroid Plexus of the Mouse from Early Development and into Adulthood While Claudin-5 is Restricted to Endothelial Cells

**DOI:** 10.3389/fnana.2016.00016

**Published:** 2016-02-22

**Authors:** Alexandra Steinemann, Isabel Galm, Sophorn Chip, Cordula Nitsch, Ireneusz Piotr Maly

**Affiliations:** Department of Biomedicine, Section of Functional Neuroanatomy, University of BaselBasel, Switzerland

**Keywords:** blood-brain barrier, claudin-5, tight junctions, zonula occludens, occludin, ependyma, alkaline phosphatase

## Abstract

A primary function of epithelial and endothelial monolayers is the formation of barriers that separate tissues into functional compartments. Tight junctions (TJs) seal the intercellular space between the single cells of a monolayer. TJs thus contribute importantly to the homeostasis of the cerebrospinal fluid as they help in maintaining the blood-brain barrier (BBB) and the blood-cerebrospinal fluid barrier (CSF). The composition of TJs differs by its localization as well as the stage of development according to its respective function. Claudin-3 is typically present in the epithelia and has been claimed to be a constituent of the BBB. It is, however, notoriously difficult to demonstrate its expression in endothelial cells of the brain vasculature at the morphological level by means of immunohistochemical techniques. Using an improved fixation strategy (4% paraformaldehyde at pH 11, in the presence of EDTA) and the sensitive alkaline phosphatase as a detection system, we show that claudin-3 is present in mouse epithelia from embryonic day 14 onwards. In brain, it is restricted to the anlage of choroid plexus in the ventricles, together with claudin-1 and -2. In adult mice, it is clearly delineating the epithelium of the choroid plexus in the lateral and fourth ventricles. In contrast, in cerebral blood vessels claudin-3 as well as claudin-1 and -2 are absent in cerebral blood vessels during all developmental stages up to adulthood. Rather, the BBB is characterized by the presence of claudin-5, ZO-1 and occludin. Thus, in mice claudin-3 is an important constituent of TJ in the embryonic and in the adult choroid plexus.

## Introduction

The central nervous system (CNS) is tightly sealed from the fluctuating composition of the blood by the blood-brain barrier (BBB) and the blood-cerebrospinal fluid (CSF) barrier (BCSFB). The BBB and BCSFB can be localized to highly specialized brain microvascular endothelial cells and choroid plexus epithelial cells (CPEC), respectively. Besides its barrier function, CPECs have a secretory function and produce the CSF.

**Figure 1:**
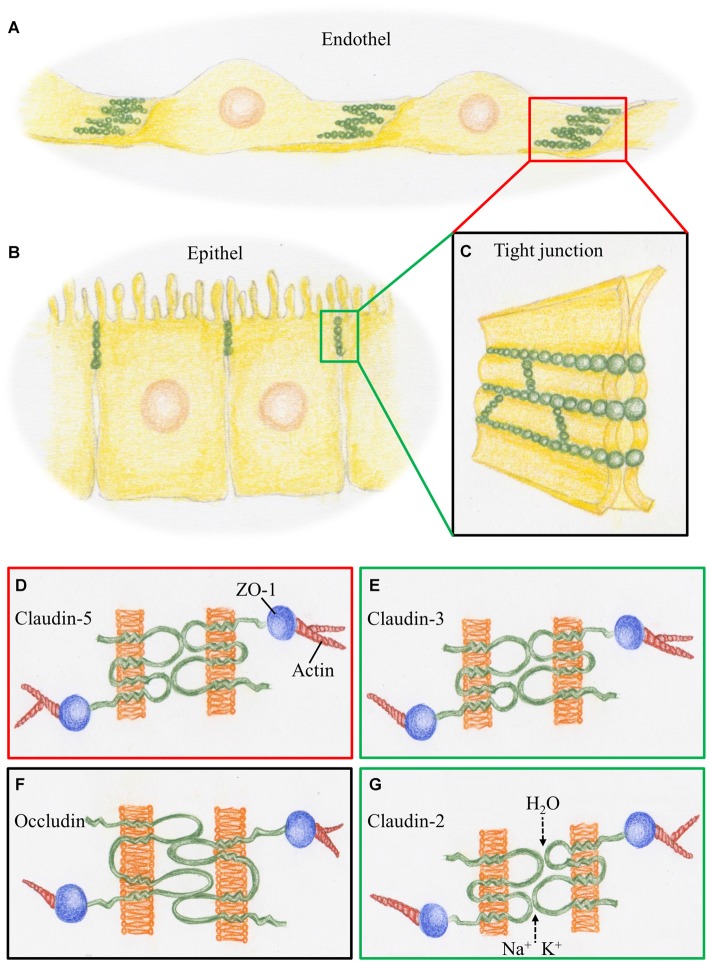
**Schematic representation of tight junctions (TJs) constituting the blood-brain barrier (BBB) and the BCSFB. (A)** At the cerebral blood vessels TJ strands (green) are arranged at the overlapping parts of neighboring endothelial cells. **(B)** At the choroid plexus, TJ strands are arranged at the apical parts of the epithelial cells. **(C)** TJ strands connecting neighboring cell membranes prevent intercellular transport. **(D)** The BBB, represented by the red box, is characterized by claudin-5, a phosphoprotein with four transmembrane domains. It is linked via the scaffolding protein ZO1 to proteins of the cytoskeleton such as actin. **(E)** The BCSFB, represented by the green box, is characterized by claudin-3, which acts as a sealant, as does claudin-5. **(F)** Occludin, a tetra span transmembrane protein with two large extracellular loops is a obligatory member of TJ strands, as presented by the black box. **(G)** Claudin-2, a member of the TJs in BCSFB, is a paracellular H_2_O and cation channel.

The BBB is composed of a monolayer of endothelial cells surrounded by a basal membrane, pericytes and perivascular astrocytes (e.g., Haseloff et al., 2005). Highly specialized cerebral endothelial cells (CECs) form a tight seal but also mediate the selective transcellular transport of nutrients and other essential components into the brain, and the efflux of potentially toxic metabolites from the nervous tissue. CECs allow little nonspecific transendothelial transport since they exhibit very low pinocytotic activity (e.g., Brightman and Reese, 1969; Nitsch et al., 1986), controlled by a transmembrane protein named Mfsd2a, a member of the major facilitator superfamily, which is exclusively expressed in CECs (Ben-Zvi et al., 2014). The paracellular route between individual CECs is sealed by an elaborate network of complex tight junctions (TJs) that interconnects endothelial cells. The morphological correlate of the BCSFB is found at the level of the CPEC, where TJs seal its apical borders against the CSF (for review, see Vorbrodt and Dobrogowska, 2003; Wolburg and Paulus, 2010). In Figure 1 the position of the barriers in CEC and CP are schematically represented and their molecular make-up shown as detailed in the following paragraphs.

TJs are formed by occludin and claudins, transmembrane proteins linked to the cytoskeleton through interactions with accessory proteins, i.e., zonula occludens (ZO-1), -2, and -3. ZO proteins act as a scaffold for multiple intracellular signaling pathways and are involved in regulation of TJs function (for review, see Hawkins and Davis, 2005). Occludin was the first integral membrane protein described to be exclusively localized within TJs including the BBB (Furuse et al., 1993) and to regulate size-selective paracellular diffusion of hydrophilic molecules (Balda et al., 2000). However, in mice carrying a null mutation of the occludin gene TJ strands formed normally (Saitou et al., 2000) suggesting a non-essential role for occludin in TJ formation. By contrast, transfection experiments with TJ-free fibroblasts have demonstrated that claudins, which comprise a gene family of at least 27 members of integral membrane proteins (Mineta et al., 2011), can reconstitute membranous strands similar to those observed in epithelial cells (Furuse et al., 1998). Removal or addition of claudins generally does selectively affect the barrier functions of TJs for size and charge. Specific claudins act as sealants, others form paracellular ion-selective channels (Amasheh et al., 2002). Furthermore, claudins show a tissue-specific expression pattern, with a unique combination of claudins that determine the paracellular tightness and ion-selectivity (for recent reviews, see Bauer et al., 2014; Markov et al., 2015).

In brain, claudin-5 was identified as a critical regulator of capillary endothelial cell permeability (Nitta et al., 2003). The BBB of claudin-5-deficient mice was permeable in a size-selective manner. Other claudins, which have been reported in CECs include claudin-1 (Liebner et al., 2000), claudin-3 (Wolburg et al., 2003) and claudin-12 (Nitta et al., 2003). Claudin-1, -2 and -11 together with occludin were described in the CPEC (Wolburg et al., 2001). However, these observations were challenged by several authors: Kominsky et al. (2007) reported that claudin-3 was undetectable in CNS tissues with the exception of the CPEC and Ohtsuki et al. (2008) reported that in contrast to claudin-5, the mRNA expression levels of claudin-1 and -3 in mouse brain capillary endothelial cells were low and the involvement of these claudins in TJ formation at the BBB was likely to be minor.

CECs as well as CPECs play an important role in homeostasis and composition of the CSF. During development, requirements for protein and fluid transport fluctuate considerably and this is governed by the CP and its barrier (Liddelow, 2015). Recent data show that in rats, CPECs are characterized by claudin-1 (Ghersi-Egea et al., 2015) and claudin-3 (Kratzer et al., 2012). Similar data in mice are still lacking.

Knowledge of the development and molecular composition of cerebral barriers is of major clinical relevance under several aspects. For example, parasites such as trypanosomes interact with claudin-1 and -11 at the CPEC resulting in infections of the CNS (Mogk et al., 2014). In multiple sclerosis (MS) two different impairments of both CNS barriers are seen: blood-borne molecules entering the brain due to BBB leakage and the active migration of inflammatory cells into the CNS (Coisne and Engelhardt, 2011). In the case of cancer, the trafficking of metastatic cancer cells into the brain is under control by CNS barriers (Harhaj and Antonetti, 2004). On the other hand, permeabilization of CNS barriers would allow the treatment of intracerebral tumors (Deeken and Löscher, 2007). Finally, the observation that certain claudins are highly expressed in certain cancers such as claudin-1 in melanomas (Leotlela et al., 2007), and claudin-3 and -4 in ovarian, breast, lung and kidney tumors (Kominsky et al., 2004; Agarwal et al., 2005; Hewitt et al., 2006) offers the possibility to target the tumors with anti-claudins. Anti-claudins could, however, if systemically applied interfere with vital barriers, as the BBB and BCSFB warranting the need for a better understanding of their molecular characteristics.

TJ proteins are difficult to localize immunocytochemically. They are concentrated in cell membranes and confined to the narrow border that lies between neighboring cells. If the structure is, in addition, convoluted, as in the case of cerebral microvessels, only tiny parts of the membranes are exposed in thin sections and detection becomes even more difficult. This problem can be overcome by using whole-mounts of brain vessels as in organotypic slice cultures, e.g., (Bendfeldt et al., 2007), or 3D reconstruction in thick slices (Paul et al., 2013). A disadvantage of these approaches is that only limited parts of the tissue can be scrutinized. Differences in TJ proteins have been analyzed concerning the different portions of the vascular tree (Paul et al., 2013). Possible differences in the molecular composition of TJs in single brain regions have not yet been demonstrated in detail. Only a very sensitive immunohistochemical staining technique on thin sections of whole brain might reveal variations in protein expression.

In view of the importance of determining the precise tissue localization of TJ proteins in brains of the adult and developing mouse we aimed to develop a sensitive immunohistochemical staining protocol which allows the study of the localization of TJ proteins in frozen sections of whole brains. We show here that, in addition to claudin-1 and claudin-2, claudin-3 is present in CPECs but absent in CECs. Preliminary data from this study were presented in Nitsch et al. (2011).

## Materials and Methods

Animal experiments were carried out in accordance with the European Communities Council Directive of 24 November 1986 (86/609/EEC) and were reviewed and permitted by Swiss authorities and surveyed by the cantonal veterinary office (project 2064). C57BL/6J mice from embryonic day (E) 14 to adult were used. Embryos (E14, E16 and E18) were collected from mothers that had been killed quickly by decapitation. Adult and postnatal day (P) 2, P4, P6, and P10 mice were decapitated. All animals entering this study were unperfused since we had observed in previous studies that perfusion impaired the maintenance of the barrier markers (Steinemann and Nitsch, unpublished observation). Whole heads (from E14 to P6 mice) were frozen in isopentane (Sigma-Aldrich, St. Louis, MO, USA) at −50°C. From P10 and adult animals, brains were rapidly removed from the skull, directly embedded in Tissue-Tek and snap-frozen in isopentane at −50°C and stored at −20°C. Fifteen micrometer (μm) thick sections of unfixed tissue were obtained at a cryostat temperature of −24°C and placed on Super Frost Plus slides (Menzel-Glaeser, Braunschweig, Germany) and dried over night at room temperature.

To enhance the detection sensitivity for immunoreactions we used alkaline phosphatase for detection and modified the fixation protocol in order to eliminate endogenous phosphatase activity. Mounted sections were immersion-fixed at pH 11.0 in 4% paraformaldehyde containing 20 mM Na-EDTA for 1 h at RT, followed by defatting/permeabilization through graded methanol in Imidazole Buffered Saline (IBS) consisting of 20 mM imidazole in 0.9% NaCl at pH 7.4 (100% methanol for 30 min, 80% methanol/20% IBS for 10 min, 60% methanol/40% IBS for 10 min).

Afterwards, sections were immunoblocked with 3% bovine serum albumin (BSA), 1% normal goat serum, 0.25% methylamine, 0.2% Triton X-100 in IBS for 30 min. Primary and secondary antibodies were diluted 1:100 in the same solution. Primary antibodies (rabbit polyclonal anti-claudin-1, -claudin-2, -claudin-3, -claudin-5, -occludin and -ZO-1) from Invitrogen-Zymed, Switzerland and anti-laminin from Sigma-Aldrich were incubated overnight at 4°C. Following washes in IBS, sections were incubated with goat anti-rabbit IgG conjugated with alkaline phosphatase (Jackson ImmunoResearch, Roche, Switzerland) as secondary antibody for 2 h at room temperature. Immunoreaction was visualized with a BCIP/NBT alkaline phosphatase substrate system (0.57 mM BCIP, 0.30 mM NBT) in AMPD buffer (5 mM MgCl2, 100 mM NaCl, 50 mM 2-Amino-2-methyl-1,3-propanediol adjusted to pH 9.5 with 1 M HCl) at room temperature for 10 min.

For controls, the primary antibody was omitted. In addition, the specifity of antibodies was tested on homogenates of mouse brain or rat liver using our high-resolution SDS electrophoresis system (Maly and Nitsch, 2007; Maly and Landmann, 2008). For detection of endogenous activity of alkaline phosphatase, cryosections were fixed in 4% paraformaldehyde in PBS at pH 7.4 for 30 min at room temperature, rinsed with AMPD buffer and incubated with BCIP/NBT alkaline phosphatase substrate system for 10 min.

After washing with PBS for 5 min and dehydration in ethanol for 10 min, sections were coverslipped in Mowiol and viewed on a Nikon Eclipse E800 microscope equipped with a ProgRes C 14 plus camera (Jenoptik, Jena, Germany). Pictures were taken using the image capture software ProgRes Capture Pro 2.5.

## Results

**Figure 2:**
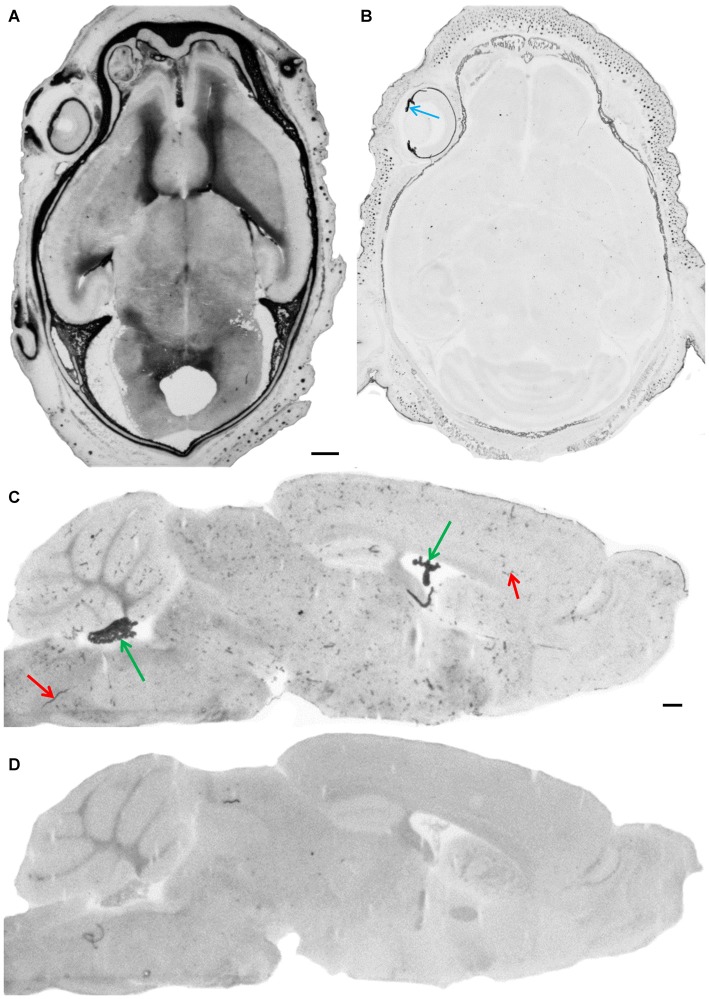
**Endogenous alkaline phosphatase activity and its blockage.** After fixation of cryosections under standard conditions (4% paraformaldehyde/PBS pH 7.4) intense staining of AP in meninges and in the parenchyma of developing brain is shown in a horizontal section through the whole head of an E18 embryo **(A)**. In the adult brain, AP staining is observed in choroid plexus (green arrows) and in blood vessels (red arrows) as shown here in a parasagittal section **(C)**. Cryosections under alkaline fixation condition in the presence of EDTA (4% paraformaldehyde/20 mM Na-EDTA, pH 11.0), completely inactivates endogenous alkaline phosphatase activities in the embryonic head **(B)**, where only pigmented structures such as the iris—see blue arrow—and the choroid of the eye bulb give a dark appearance and in the adult brain **(D)**. Scale bar = 0.5 mm in **(A)** applies also for **(B–D)**.

In the present study, a BCIP/NBT alkaline phosphatase based immunohistochemistry staining protocol was used to probe for the presence of TJ proteins in frozen sections of the developing and adult mouse brain. To block endogenous alkaline phosphatase activity, sections were fixed in 4% paraformaldehyde/Na-EDTA solution at pH 11.0. Our initial experiments showed that, in fact, at pH 11, adequate fixation of brain sections resulted in good structural preservation of the nervous tissue. At this high pH, paraformaldehyde in combination with EDTA completely inactivated endogenous alkaline phosphatase activities in whole heads of embryonic mice and in brains of adult mice (Figure 2). This pretreatment did not impair immunoreactivity of common markers as shown here for laminin, a marker for the basal lamina (see Figure 4C). This allowed us to use the highly sensitive alkaline phosphatase detection method to visualize the presence of TJ proteins.

**Figure 3:**
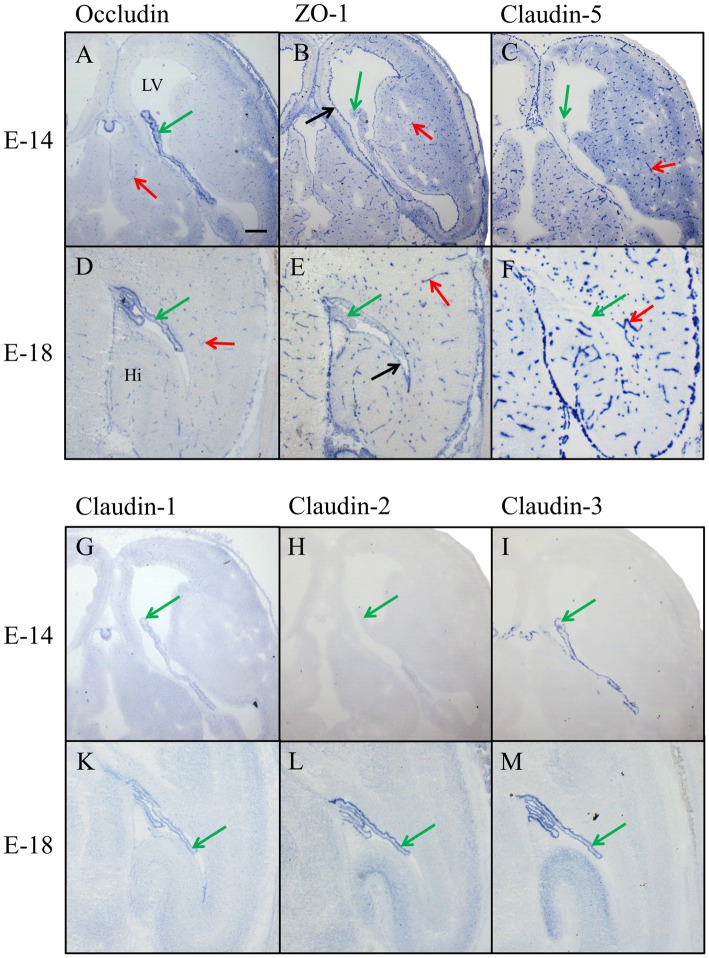
**Immunohistochemical stainings of horizontal cryosections of the developing brain (E14 and E18).** Occludin immunoreactivity **(A,D)** is present in brain vessels and in epithelium of choroid plexus and ZO-1 **(B,E)** in brain vessels and in the neuroepithelium of the ventricle. Claudin-5 immunoreactivity **(C,F)** is selectively present in the endothelium of brain vessels. Anti-claudin-1 **(G,K)**, anti-claudin-2 **(H,L)** and anti-claudin-3 **(I,M)** do not stain any blood vessels but the epithelium of the choroid plexus. Immunoreactivity for claudin-3 is strong from E14 onward, for claudin-1 faint at E14 and comparable with claudin-3 at E18, and for claudin-2 it only appears at E18. LV, lateral ventricle; Hi, Hippocampus, green arrow choroid plexus, red arrow blood vessel, black arrow ependyma. Scale bar = 100 μm in **(A)** applies also for **(B–M)**.

In the prenatal brain, occludin immunoreactivity was found in intraparenchymal blood vessels, in the meninges and in the choroid plexus but not in the ependyma for E14 (Figure 3A) and E18 (Figure 3D). ZO-1 was present in CECs, the neuroepithelium of the ventricles (i.e., the choroid plexus and ependyma), and in the meninges covering the surface of the brain (Figures 3B,E). Of the claudins tested, only claudin-5 showed strong selective immunoreactivity in CECs (Figures 3C,F); localized along intracerebral and pial vessels that were on their way to the brain. Claudin-1, -2, and -3 were absent from endothelial cells but could be detected in the epithelium of the choroid plexus. Strong immunoreactivity was present for claudin-3 from E14 onward in the choroid plexus (Figures 3I,M), while claudin-1 was faintly stained (Figure 3G) and claudin-2 was absent (Figure 3H) at the early embryonic stages. At E18, additionally to claudin-1 and -3, immunoreactivity for claudin-2 was detected in the choroid plexus and at this stage of development, the staining intensity of claudin-1, claudin-2 and claudin-3 were similar (Figures 3K–M).

**Figure 4:**
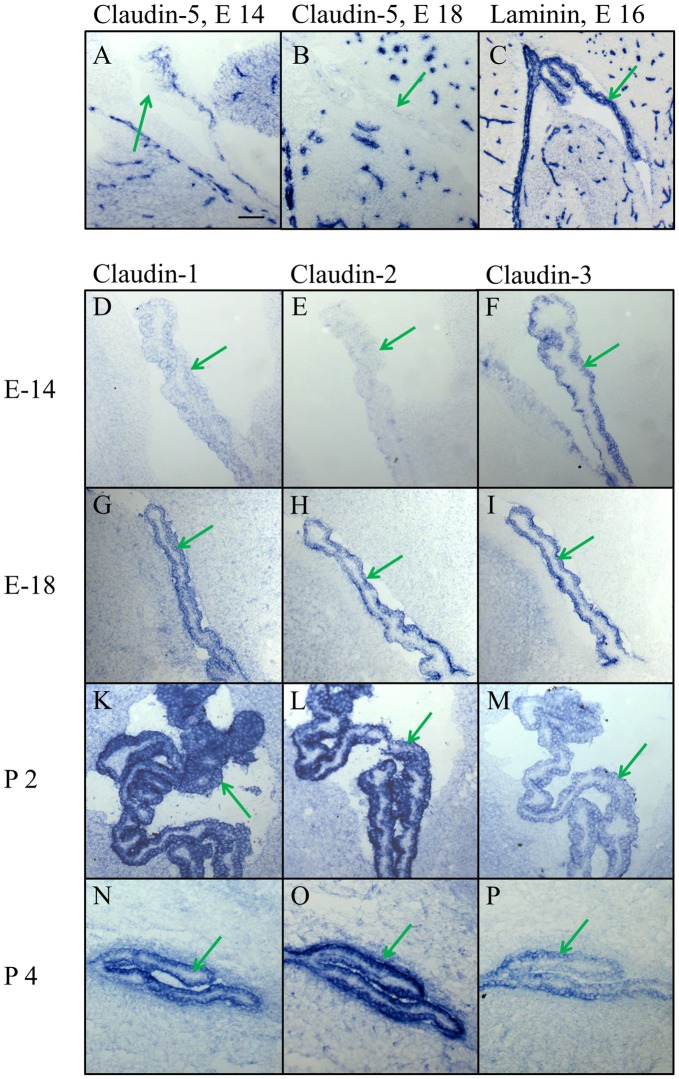
**High magnification of the choroid plexus in the lateral ventricle during embryonic and early postnatal development. (A,B)** Claudin-5 heavily stains the blood vessels in the brain parenchyma and in the choroid plexus, but not the epithelium of choroid plexus. **(C)** The marker for the basal lamina, laminin, is outlining the border between CPECs and CP parenchyma, as well as the borders of the intracerebral vessels. **(D–P)** Claudin-1, claudin-2 and claudin-3 are expressed in epithelial cells of the choroid plexus with varying density at different developmental stages. Endothelial cells of blood vessels, however, never express claudin-1,-2 or -3. Green arrow choroid plexus. Scale bar = 100 μm in **(A)** applies also for **(B–P)**.

**Figure 5:**
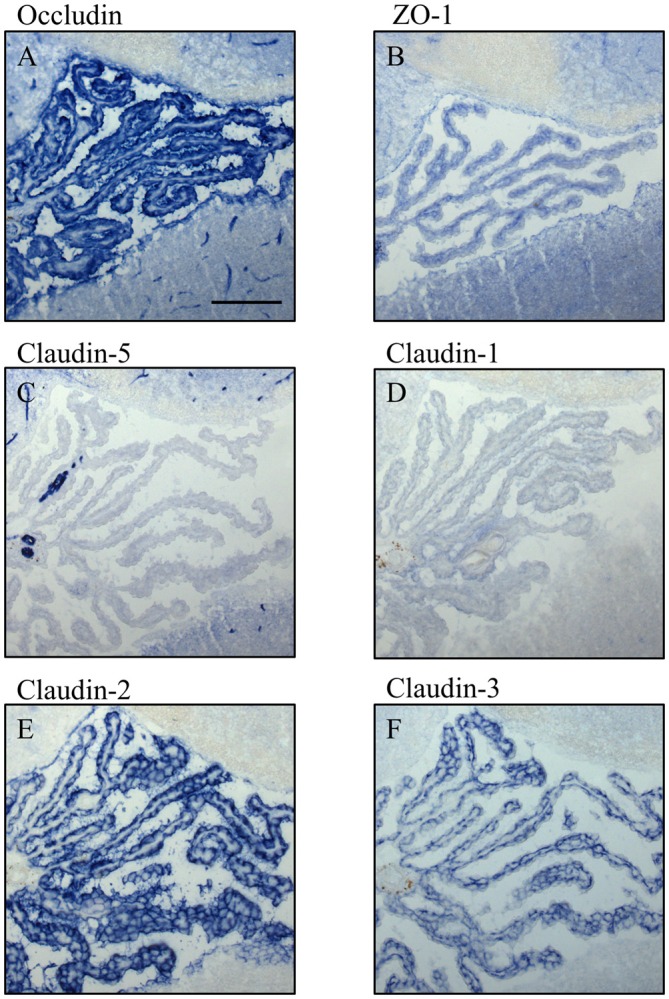
**Immunocytochemistry of choroid plexus in the fourth ventricle on sagittal cryosections of adult mouse brain.** Immunoreactivity for occludin **(A)** is present in blood vessels and in the apical parts of the plexus epithelium as well as the ependyma. ZO-1 **(B)** immunoreactivity is relatively faint in the adult and labels blood vessels, basal parts of the plexus epithelium and the ependyma. Claudin-5 **(C)** is selectively and strongly labeling brain blood vessels. It is absent from the plexus epithelium and the ependyma. Immunoreactivity for claudin-1 **(D)**, Claudin-2 **(E)** and claudin-3 **(F)** is absent from the brain vessels. The immunoreactivity against claudin-1 in CP is weak compared to immunoreactivities against claudin-2 and -3. Scale bar = 100 μm in **(A)** applies also for **(B–F)**.

**Figure 6:**
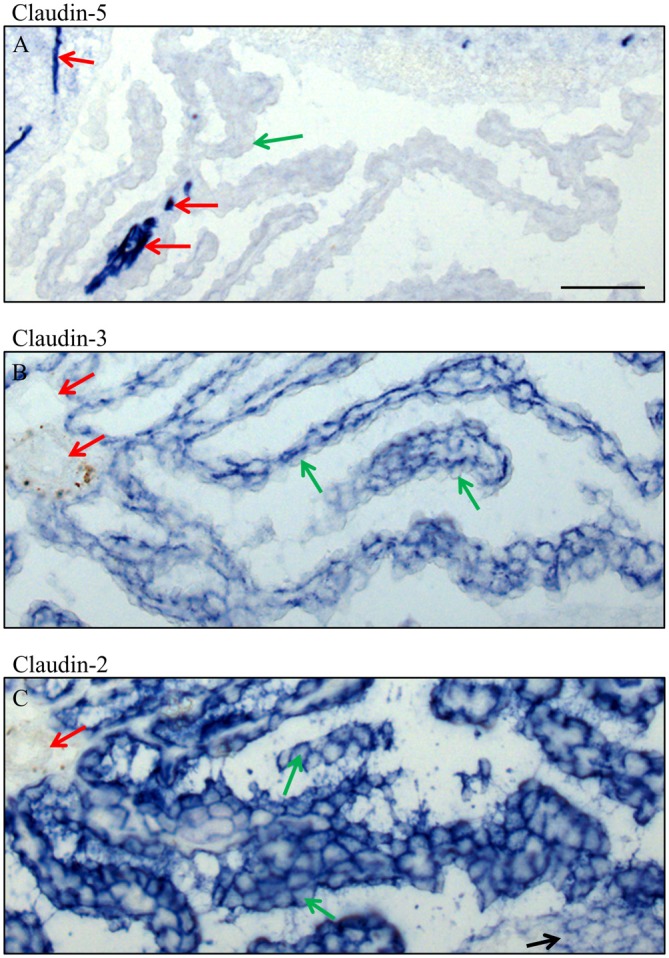
**High magnification of immunocytochemistry of choroid plexus in the fourth ventricle on sagittal cryosections of adult mouse brain.** Immunoreactivity for claudin-5 **(A)** is only present in blood vessels, in intracerebral vessels as well as in an occasional intraparenchymal vessel of the choroid plexus. Claudin-3 **(B)** is restricted to the epithelium of choroid plexus and claudin-2 **(C)** is in the epithelium of the choroid plexus and the ependyma. Green arrow choroid plexus epithelial cell, red arrow endothelium of blood vessels, black arrow ependyma. Scale bar = 50 μm in **(A)** applies also for **(B,C)**.

In high magnification of the choroid plexus it became evident that claudin-5, if at all present, was situated in blood vessels of the plexus parenchyma and not in the plexus epithelium (Figures 4A,B), in contrast to claudin-1, -2, and -3. The analysis of early development from E14 to P4 showed a characteristic time pattern of expression for each of these claudins. The immunoreactivity of claudin-3 remained relatively constant during all ages and with all batches tested (Figures 4F,I,M,P), while claudin-1 and -2 gave more variable results. Claudin-1 was present at E14 (Figure 4D), then increased in expression during development (Figure 4G), peaked around birth (Figure 4K) and declined towards adulthood (Figure 4N). Claudin-2 was not yet detectable at E14 (Figure 4E), present at E18 (Figure 4H) and exhibited at postnatal age a massive increase (Figures 4L,O) that persisted during further development at P6 and P10 (not shown) well into the adult brain (Figures 5E, 6C).

In adult brain (Figures 5, 6), claudin-2, and -3 were clearly detectable in the epithelium of the choroid plexus. Neither claudin-1, -2 or -3 antibodies stained CECs. As previously demonstrated by others, claudin-5 was present in endothelial cells of the cerebral vasculature and occasionally in endothelial cells of the choroid plexus but not in its epithelium (Figures 5C, 6A). Occludin (Figure 5A) and to a lesser extent ZO-1 (Figure 5B) were expressed in CEC and the epithelium of the choroid plexus. Claudin-3 was restricted to the junctions between the epithelial cells of the choroid plexus (Figures 5C, 6A) and claudin-2 was found not only between the CPEC but outlining the neuroepithelial cells of the ependyma (Figures 5E, 6C).

## Discussion

Based on the cellular structure of barriers, be they epithelial as in the case of the BCSFB or endothelial as in the case of the BBB, presence of TJ proteins is difficult to verify under compound light microscopy. They form narrow lines between neighboring cells and in case of the convoluted intracerebral vessels, only small parts of the structure are present in thin sections. As a result of this low antigen content the use of a detection system that is more sensitive than immunofluorescence or the horseradish peroxidase detection systems is necessary. Such a system exists in the form of the BCIP/NBT alkaline phosphatase based immunohistochemical staining protocol (De Jong et al., 1985) which due to the absence of H_2_O_2_ has the advantage of being more gentle, so that development can be carried out over hours if necessary. This alkaline phosphatase staining system has been adapted here to probe for the distribution of TJ proteins in frozen sections of unperfused developing and adult brains.

To block endogenous alkaline phosphatase activity, sections were fixed in 4% paraformaldehyde/Na-EDTA solution at pH 11.0. This basic fixation pH was recommended for the localization of tyrosine hydroxylase in the dopaminergic system of rat brains (Berod et al., 1981). Our initial experiments showed that in fact, at pH 11.0, adequate fixations of brain sections were obtained. At this alkaline pH, paraformaldehyde in combination with EDTA completely inactivated endogenous alkaline phosphatase activities in brain and bone. Immunoreactions for well-documented tissue markers such as GFAP (astrocyte marker, not shown) and laminin gave results indistinguishable to other fixation protocols. Using this immunohistochemical staining protocol, we could confirm previous data on the molecular make-up of the BBB during development and in the adult rodent (see Ballabh et al., 2004; Abbott et al., 2010): occludin, ZO-1 and claudin-5 were expressed in CECs from early embryonic age on into adulthood. No clear differences between brain regions were found. While occludin and to a lesser extent ZO-1 were present in the epithelia of the BCSFB, claudin-5 was found exclusively in blood vessels, occasionally also labeling vessels in the stroma of CP, but never in the epithelium. Claudin-5 is responsible for the size-selective barrier at the BBB (Nitta et al., 2003; Coisne and Engelhardt, 2011) playing a key role in BBB development and maintenance as well as being lethal by the lack of it (Coisne and Engelhardt, 2011).

Neither claudin-1 nor -2 and -3 were detectable in cerebral vessels, but from the early embryonic age on in CPECs. Claudin-1 and -2 had previously been detected in the choroid plexus (Lippoldt et al., 2000), but claudin-3 has been referred to as a key molecule of the TJ of the BBB and considered to be responsible for their tightness (Wolburg et al., 2003). Recent data in humans and mice suggest, however, that claudin-3 is undetectable in CNS tissue with the exception of the CP (Kominsky et al., 2007; Ohtsuki et al., 2008). Similar to our findings, Kooij et al. (2014) showed a restricted protein expression of claudin-3 in CPECs with a typical TJ strand arrangement in adult mouse and human brain tissue. Also, Kratzer et al. (2012) demonstrated this for claudin-1, -2 and -3 in rat CPECs.

Throughout development the immunoreactivity patterns of claudins at the choroid plexus underwent marked changes. At E14, claudin-3 was most prominent at CPECs while claudin-2 immunoreactivity was absent. At E-18, a similar staining intensity of claudin-1, -2 and -3 were observed. In adult brain, however, immunoreactivity of claudin-1 was diminished and immunoreactivity of claudin-2 was strong in CPECs. In contrast, the immunoreactivity of claudin-3 in CPECs remained relatively constant during all ages tested. Kratzer et al. (2012) demonstrated by rtPCR and Western blot in the developing rat brain and by immunohistochemistry in pre- and postnatal human brain the expression of claudin-1, -2 and -3 at CPECs. Similar to our observations, they found that claudin-1 is upregulated at embryonic stages and that claudin-2 was continuously increasing with age. In rats, claudin-3 was detected at all stages but down-regulated in adult animals. However, in mice as shown in the present study there was a strong immunoreaction for claudin-3 in all ages. These divergent findings might be explained by the use of Western blot and rtPCR, in comparison to immunohistochemistry.

Overall, the composition of the CSF-barrier seems to be dynamic and constantly changing during the development, likely reflecting the different demands of the developing brain for proteins and fluid volume and composition (Ghersi-Egea et al., 2015; Liddelow, 2015). Thus, claudins-1 and -3 with sealing function behave differently from claudin-2 which functions as water and cation channel (Yu et al., 2009; Rosenthal et al., 2010). For MS, understanding the contribution of the different claudins in neuroinflammation and the role of claudin-3 in BCSF (Kooij et al., 2014) is of relevance.

Finally, besides the superior sensitivity, the immunocytochemical technique presented here has an additional advantage: whole brain sections can be analyzed at high and low magnification to study regional variation in protein expression. While we did not observe major differences in the expression of TJ proteins between brain regions in normal healthy brain, this might be quite different under experimental conditions and in the diseased brain from stroke to MS. Furthermore, claudin-3 and -4 were identified as receptors for cytotoxic *Clostridium perfringens* enterotoxin (CPE, Katahira et al., 1997). The ability of CPE to rapidly and specifically lyse cells expressing claudin-3 and/or -4 might be useful for the treatment of tumors expressing these claudins (see Black et al., 2015). However, an important concern for any new drug therapy is systemic toxicity, since many other tissues express claudins. Determining the presence or absence of claudins is invaluable, especially on their localization at the barriers of the brain and can now be reliably tested using the present method.

## Author Contributions

IPM and CN conceived the project and designed the single steps of its implementation; AS and IG carried out the experimental work, AS and IPM analyzed the data and SC helped in the interpretation. All authors discussed the results and were involved in drafting the article. The manuscript was written by IPM and CN with important contributions from SC. All authors have approved the final version of the manuscript and agree to be accountable for all aspects of the work, in particular concerning questions related to the accuracy and integrity.

## Conflict of Interest Statement

The authors declare that the research was conducted in the absence of any commercial or financial relationships that could be construed as a potential conflict of interest.
